# Acute Hepatitis of Unknown Origin (AHUO)—The Puzzle Ahead

**DOI:** 10.3390/diagnostics12051215

**Published:** 2022-05-12

**Authors:** Consolato M. Sergi

**Affiliations:** Division of Anatomic Pathology, Children’s Hospital of Eastern Ontario, Ottawa, ON K1H 8L1, Canada; csergi@cheo.on.ca; Tel.: +1-613-737-7600 (ext. 2427); Fax: +1-613-738-4837

An intriguing form of hepatitis has been detected in more than a hundred children worldwide. Most often seen in the United Kingdom, United States, Spain, and Ireland, the number of patients affected may in fact be a thousand or more. The alarming and challenging wave of cases involves patients typically in the second infancy in the age group of 1 and 5 years, but adolescents and adults have been reported. In a few of these patients, liver transplantation was necessary. In the United Kingdom, most of the cases are in England, while in the United States, the states of Alabama and North Carolina have observed most of the cases, at least initially. It does not seem that there is a uniformity of findings because, in some patients, a viral origin has been suggested. In others, either a metabolic-genetic origin or autoimmune pathogenesis has been indicated. On 15 April 2022, the World Health Organization (WHO) stated that there is an absolute priority to identify the etiology of these cases to promote a prompt clinical and public health action.

Hepatitis is an inflammation of the liver, which can involve either the portal tract or the liver acinus, or combine both phenotypes. The causes of hepatitis are multi-etiologic. Of note, the exclusion of a hepatitis virus, A, B, C, D, or E, is at first tackled, not only in clinics but also in laboratory medicine. Liver damage can also be an event following a toxin, such as drugs or botanicals. Other events may include an excessive accumulation of fat in the hepatocytes, or NAFLD (non-alcoholic fatty liver disease)/NASH (non-alcoholic steato-hepatitis), or autoimmunity/immunologic dysregulation. The clinical phenotype of the patients harboring the acute hepatitis of unknown origin includes gastrointestinal symptoms with abdominal pain, diarrhea, and vomiting as prodromal signs, which are followed by a picture of severe acute hepatitis with jaundice and an increase of liver enzymes (AST, aspartate transaminase, and ALT, alanine aminotransaminase). Both AST and ALT are typically in the range of 500 IU/L or greater. In many patients, there is no fever, and in all patients, the A through E viral etiologies have been excluded. Moreover, travel into a country with endemic liver disease has not been recorded, with most of the cases being autochthonous. In at least 74 cases, an association with adenovirus infections has been detected, and 18 cases have been labeled as F type 41. SARS-CoV-2 was detected in 20 children of those that have been tested, while in 19 children, both adenovirus and SARS-CoV-2 were detected [1].

Adenovirus is rarely associated with liver disease. There may exist a weak immune status as potential life condition in many cases with adenovirus infection, as demonstrated by McKillop et al. in a patient with atypical teratoid rhabdoid tumor [2]. If adenovirus is a hypothesis for the AHUO, it does not fully clarify the severity of the clinical phenotype. The infection with adenovirus type 41 has not been associated with such a clinical picture. In fact, an immunocompetent individual does not react to the severe disease when encountering an adenovirus, in which most of the cases are linked to respiratory symptomatology. Adenovirus infections are generally self-limited infections in an immunocompetent individual. These infections are challenging to explain AHUO, and a simultaneous or coincidental event cannot be ruled out. Adenoviridae members are usually associated with upper respiratory infections, gastroenteritis, and urinary system infections. Adenovirus type 41 is usually not typically associated with hepatitis, being in no cases an etiology of hepatitis in otherwise healthy infants or children. There is the consideration that this virus and SARS-CoV-2 (severe acute respiratory syndrome–related coronavirus 2) may establish a line of attack against hepatocytes. However, other infections and, possibly, no infections need to be ruled out. The immediate exclusion of a potential etiology of COVID-19 vaccination for this AHUO in some media is appalling. The mRNA vaccines have also been used in some adolescents and young adults in several countries. In this extensive case search, we cannot rule out that the mRNA vaccines and the prolonged use of face masks in children may have undermined the immune system of some children. An extended use of face masking is not only deleterious for children’s mental health, but also for their immune system due to deficient antigen exposure. Geographical and temporal information needs to be gathered and properly assessed by institutions and individuals without a conflict of interest. The possibility of a link between AHUO and mRNA vaccines, the type of vaccines typically given to this age group, needs to be fully and thoroughly explored. COVID-19 vaccination has the potential to trigger clinical issues, as we have seen regarding myocarditis in several reports. The aberrant immunologic response to the vaccines may lead to many health issues, including hepatitis.

Vaccination is a strategic key in the fight against the COVID-19 pandemic. It has promoted the evolution of a pandemic to endemic in several countries and eradicated several lethal microorganisms in many countries [3]. Nevertheless, several reports have associated autoimmune hepatitis-like conditions with COVID-19 vaccines. It remains crucial that all etiologies should be rigorously explored without bias at this stage. It may be coincidental, reflect a transient drug-induced liver injury (DILD) [4,5,6], or even involve a SARS-CoV-2-triggered antigenic-specific immunologic activation. In adult patients presenting with COVID-19-vaccine-related etiology, a highly activated cytotoxic CD8 T cellular infiltrate, including the SARS-CoV-2-specific T cell population, harbors the CD8 antigen correlated with a peripheral activation SARS-CoV-2-specific T cells carrying the CD8 antigen (cytotoxic T lymphocytes) [7,8,9,10,11,12,13,14,15,16,17,18,19,20,21,22,23,24,25,26,27,28,29,30,31]. It suggests that post-COVID-19 vaccination hepatitis involves a vaccination-elicited antigen-specific immunologic response. This picture is similar to the histologic picture of hepatitis A infection or autoimmune pathogenesis.

In conclusion, our line of action needs to keep an open mind to consider any option still open on the table, including COVID-19 vaccination. Proper documentation with serology, electron microscopy, and molecular analysis for all patients is critical. It is important to consider wisely at this time the cost–benefit relationship for this kind of vaccination and prolonged face masking in children and youth. We may consider limiting such vaccines to patients affected with co-morbidities. The multi-system inflammatory syndrome is dreadful, but remains an infrequent complication of COVID-19.
